# The Rev. G. E. Aspinall's Speech

**Published:** 1912-02-17

**Authors:** George E. Aspinall

**Affiliations:** Royal Halifax Infirmary


### The Editor's Letter Box.

### THE REV. G. E. ASPINALL'S SPEECH.

*To the Editor of* The Hospital.

Sir,—I do not propose to take any notice of the somewhat incoherent and certainly extremely discourteous observations which you thought fit to make in your last issue of The Hospital concerning me, and the remarks I ventured to make at the annual meeting of this Infirmary. On the matter of fact, however, I should be obliged if you would allow me emphatically to deny that on that occasion I either sneered, or intended to sneer, at the resolutions which were carried at the meetings of the British Hospitals Association at London and Manchester. I voted for these resolutions, and, moreover, seconded one in similar terms at a meeting of the Managers of Yorkshire Hospitals at Leeds last July, and I must plead guilty to at least so much "self-complacency" as to believe that I have not yet reached such a state of "ineptitude" as to sneer, at any rate in public, at my own actions.—I am, yours, etc.,

George E. Aspinall.

Royal Halifax Infirmary, February 10.

[The answer to Mr. Aspinall's letter is to be found in the speech which, as chairman, he delivered at the recent annual meeting of the Halifax Royal Infirmary, the report of which appeared in the Halifax *Daily Guardian* of Wednesday, January 31.

"Referring to the Insurance Act, the chairman said . . . They in Halifax had watched it very carefully. He was sent to attend as a deputation the British Hospital Association's meeting in London in June, and with his friend Mr. Pollit he was sent to Manchester in November. 'We passed resolutions,' he remarked, 'sent deputations, and had an excellent lunch—(laughter)—and I don't know that we got further than that. We had another meeting in Leeds and passed other resolutions, but what became of them I don't know. (Renewed laughter.) They have gone to the limbo of past things.'"—These remarks and their effect upon his audience are perfectly clear indications of Mr. Aspinall's attitude to the resolutions at the time he made the above speech.—Ed., The Hospital.]

### THE ADDITIONS TO RADCLIFFE INFIRMARY, OXFORD.

*To the Editor of* The Hospital.

